# Apolipoprotein A5 alleviates LPS/d-GalN-induced fulminant liver failure in mice by inhibiting TLR4-mediated NF-κB pathway

**DOI:** 10.1186/s12967-019-1900-9

**Published:** 2019-05-10

**Authors:** Ya-Chao Tao, Meng-Lan Wang, Dong-Bo Wu, Chen Luo, Hong Tang, En-Qiang Chen

**Affiliations:** 1 Center of Infectious Diseases, West China Hospital of Sichuan University, No. 37 Guo Xue Xiang, Wuhou District, Chengdu, 610041 China; 2 Division of Infectious Diseases, National Key Laboratory of Biotherapy (Sichuan University), West China Hospital of Sichuan University, Chengdu, 610041 China

**Keywords:** Apolipoprotein A-V, Fulminant liver failure, Lipopolysaccharide, Endotoxemia, TLR4

## Abstract

**Background:**

Fulminant liver failure (FHF) is a serious clinical problem and liver transplantation is the major intervention. But the overall survival rate of FHF is low owing to the donated organ shortage. Apolipoprotein A-V (ApoA5) is a regulator of triglyceride metabolism and has been reported to act as a predictor for remnant liver growth after preoperative portal vein embolization and liver surgery. This study aimed to investigate the therapeutic effect of ApoA5 on lipopolysaccharide/d-galactosamine (LPS/d-GalN)—induced fulminant liver failure in mice.

**Methods:**

FHF mouse model was established using LPS/d-GalN and ApoA5 plasmid was injected by tail vein prior to LPS/d-GalN treatment. The expressions of ApoA5, toll-like receptor 4 (TLR4), myeloid differentiation factor 88 (MyD88), and nuclear factor kappa B p65 (NF-κBp65) were assessed by real-time PCR and western blotting. Serum alanine aminotransferase (ALT) and tumor necrosis factor-α (TNF-α) levels were measured using automatic biochemical analyzer. Histological assessment and immunohistochemical (IHC) staining were conducted. Survival rate after LPS/d-GalN administration was also determined with Kaplan–Meier curve. Meanwhile, the expression of ApoA5 in injured huh7 cells was tested. Cell apoptosis analysis was performed after huh7 cells were transfected with ApoA5 plasmid and stimulated with LPS.

**Results:**

The expressions of ApoA5 decreased both in injured huh7 cells and FHF mice. ApoA5 overexpression reduced cell death rate using flow cytometry. ApoA5 not only decreased the serum ALT and TNF-α levels but also attenuated hepatic damage in hematoxylin–eosin (HE)-stained liver section. The protein expressions of TLR4, MyD88 and NF-κBp65 were inhibited when ApoA5 overexpressed. But the inhibitory effect would weaken with the increasing concentration of LPS in spite of ApoA5 overexpression. Besides, ApoA5 improved liver injury in a dose-dependent manner and the survival rate in FHF mice increased with increasing concentration of ApoA5.

**Conclusion:**

ApoA5 had a protective effect against LPS/d-GalN-induced fulminant liver failure in mice within a certain range by inhibiting TLR4-mediated NF-κB pathway.

## Background

Fulminant liver failure (FHF), also known as acute liver failure (ALF), is a lethal clinical syndrome characterized by high mortality and rapid deterioration of liver function without underlying chronic liver disease [1]. The aetiology of FHF is complex and the mode of cell death is different depending on aetiology, thus different therapies may be required to promote liver repair [2]. FHF patients with either indeterminate aetiologies or identified aetiologies (virus hepatitis, drug-induced liver injury, etc.) are candidates for emergency liver transplantation (LT) and should lead to listing for emergency LT without delay. As one of the first-line treatment options, LT can improve the prognosis of patients admitted with FHF with survival rates falling within the 59–79% range [3]. However, less than 10% of listed patients received LT in Asia, and the overall mortality rate of wait-list is 45–60%, as the rapid progression of ALF provides only a very narrow window for LT [4]. Thus, exploring other effective therapies to increase the overall survival of fulminant liver failure has become a challenge for clinicians.

Apolipoprotein A-V (ApoA5) is part of regulatory gene cluster locating on chromosome 11 [5]. It is a relatively novel protein discovered through comparative analysis between human and mouse genomic DNA sequence, and has strong effect on triglyceride (TG) metabolism in spite of its low concentration in the plasma [6]. ApoA5 regulates plasma TG levels either through stimulation of lipoprotein lipase (LPL)-mediated TG hydrolysis or by binding to the low-density lipoprotein (LDL) receptor (LDLR) family [7]. The protein is primarily synthesized in the liver and it plays a key role in the physiology and pathogenicity of lipid in vivo [8]. Previous studies have proved that both ApoA5 transgenic mice [9] and hepatoma cells transfected with ApoA5 expression plasmid [10] can lead to elevated TG accumulation in the liver. Studies have also reported that ApoA5 is closely related with obesity, dyslipidemia, insulin resistance and metabolic syndrome [11, 12], and it is expected that ApoA5 can promotes hepatic TG storage and contribute the pathogenesis of non-alcoholic fatty liver disease (NAFLD).

Additionally, ApoA5 may act as an early predictor for remnant liver growth after preoperative portal vein embolization and liver surgery as plasma TG influences the process of liver regeneration and ApoA5 controls and determines TG levels [13]. In our previous study, we also found ApoA5 predicted the prognosis of hepatitis B virus-associated acute-on-chronic liver failure (HBV-ACLF) [14]. Except for the potential of predicting the outcomes of HBV-ACLF, we speculated that ApoA5 may also be a potential target for clinical intervention based on the fact that disorder of lipid metabolism can produce abundant toxic metabolites in the liver to induce and aggravate liver injury.

The pathophysiological process of FHF is complex, during which endotoxemia is an important factor leading to the development of liver failure. Lipopolysaccharides (LPS), part of the outer membranes of gram-negative bacteria, may lead to endotoxemia in patients with liver failure [15]. d-Galactosamine (d-GalN) can dramatically enhance the lethal effects of LPS by more than 1000-fold. The combined application of LPS and d-GalN to induce mice liver failure is widely used and acceptable to resemble human acute hepatic failure in clinic [16]. Thus in present study, we established a mouse model of FHF with LPS and d-GalN, aiming to investigate the therapeutic effect of ApoA5 in liver failure.

## Materials and methods

### Cell, animals and plasmid used in the study

Huh 7 cells, a human hepatoma cell line, were preserved in our laboratory. They were cultured in Dulbecco’s modified Eagle’s medium (DMEM) containing 10% fetal bovine and 1% penicillin/streptomycin under standard culture conditions (a humidified 5% carbon dioxide incubator at 37 °C). Male Balb/c mice (6–8 weeks old, weighing 18–20 g) were purchased from the Huaxi Laboratory Animal Center of Sichuan University (Chengdu, China). All mice were maintained under controlled conditions (24 °C, 55% humidity and 12-h day/night rhythm) with free access to food and water. The mice received human care under the Institutional Review Board in accordance with the Animal Protection Art of Sichuan University. After 1 week of acclimation, the mice were prepared for further study. ApoA5 overexpression plasmid (pEGF-N1-ApoA5) and negative control empty vector (pEGF-N1 vector) were designed and provided by *Heyuan Biotechnology* (OBIO, China).

### Experiment design

LPS (*Escherichia coli,* 0111:B4) and d-GalN were purchased from Sigma (St.  Louis, MO), and they were dissolved in pyrogenfree physiological saline (NS) for use. In the study, we aimed to investigate the role of ApoA5 in liver injury both in vitro (huh7 cells) and in vivo (mice). In vitro, huh7 cells were transfected with pEGF-N1-ApoA5 to verify the expression of ApoA5. And huh7 cells were exposed to LPS after transfection with pEGF-N1-ApoA5 to identify the role of ApoA5 in hepatocyte. The FHF mice mode was induced by stimultaneous intraperitoneal injection of LPS (20 μg/kg) and d-GalN (900 mg/kg) dissolved in NS. To investigate the dynamic change of ApoA5 expression in FHF mice, the mice were randomly divided into three groups (n = 3/group), and they were sacrificed at different time points (0, 4 and 6 h) after LPS/d-GalN injection and the liver tissue were harvested. The ApoA5 expression levels were assessed by western blotting.

To investigate the role of ApoA5 in liver injury in vivo, the mice were randomly divided into another three groups (n = 3/group). And mice were given NS (group A), pEGF-N1 vector (group B) and pEGF-N1-ApoA5 (group C) by tail vein three days before LPS/d-GalN administration. Eight hours after LPS/d-GalN administration, the mice were sacrificed and the serum and liver samples were harvested and stored for analysis.

To investigate the effect of ApoA5 on liver injury, different concentration of pEGF-N1 ApoA5 (5 μg, 10 μg and 20 μg) were injected through tail vein before LPS/d-GalN administration. And the mice were sacrificed and liver tissues were collected. To investigate the expression of molecules involved in TLR4-related pathway in different degrees of liver injury, d-GalN (900 mg/kg) and LPS with increasing concentration (20 μg/kg, 30 μg/kg and 40 μg/kg) were injected intraperitoneally to mice (n = 3/group). Eight hours after LPS/d-GalN administration, the mice were killed and liver samples were harvested and stored for analysis.

### Cellular transfection

After grown to 60% in six-well plates, huh7 cells were transfected with pEGF -N1 ApoA 5 or empty vector using X-tremeGENE HP DNA Transfection Reagent (Roche). After 6 h, the medium was replaced with normal medium and cells were cultured for an additional 42 h for protein expression and apoptosis assays.

### Flow cytometric analysis of cell apoptosis

Apoptotic cells were assessed by measuring phosphatidylserine using an annexin V- Fluorescein isothiocynate (FITC) apoptosis detection kit (SIGMA product) as per manufacturer’s protocol. Cells were collected and washed twice with PBS. Then cells were resuspended in 500 μL 1× binding buffer, and stained with 5 μL of annexin V-FITC conjugate and 10 μL of PI solution. After incubation for 15 min in the dark condition at room temperature, stained cells were analyzed by flow cytometry (Bio-Rad).

### Hepatic damage assessment

To assess hepatic damage, serum alanine aminotransferase (ALT) and tumor necrosis factor-α (TNF-α) were measured using automatic biochemical analyzer. For pathological analysis, liver tissue was fixed in 10% neutral-buffered formalin and then embedded in paraffn. Tissue sections were stained with hematoxylin–eosin (HE) using a standard protocol. The morphological criteria of vacuolization, swollen cytoplasm with disrupted cells and organelle membranes, and lytic nuclear changes served to determine necrosis [17].

### Immunohistochemistry

Paraffin sections were deparaffnized, rehydrated and incubated with primary antibody (1:200) overnight at 4 °C, and then incubated with biotinylated secondary antibody for 30 min. The sections were stained with diaminobenzideine (DAB), counterstained with hematoxylin, and evaluated under a light microscope and representative images are present.

### Determination of survival rate

The survival rates of the mice with different concentrations of pEGF -N1 ApoA 5 (5 μg, 10 μg and 20 μg) and increasing concentrations of LPS (20 μg/kg, 30 μg/kg and 40 μg/kg) were monitored for12h after LPS/d-GalN administration using Kaplan–Meier curve. The number of dead mice was counted every 2 h after LPS/d-GalN injection.

### Real-time quantitative PCR analysis

Total RNA was extracted from liver tissue with TRIzol Reagent according to the manufacturer’s instruction (Invitrogen, USA). Total cellular RNA was reverse-transcribed using Moloney Murine Leukemia Virus (MMLV) reverse transcriptase (Gibco BRL, USA). The expression of genes mRNA was measured by real-time PCR using Maxima SYBR green/ROX qPCR Master Mix (Fermentas Life Sciences, Canada). The primer sequences of GAPDH (the reference) and candidate genes are listed in Table 1. And the fold changes of the expression of the candidate genes relative to the reference gene were calculated using the normalized expression (ΔCt) method with default threshold values using CFX Manager Software (Bio-Rad).

**Table 1:** Primers used for real time PCR

Name	Primer
ApoA5	F: 5′TGAAAGGCAGCTTCGAGCAA3'
	R: 5′GTGCTTCGCAGCCATGTAG3'
TLR4	F:5′TGGTGTCCCAGCACTTCATC3'
	R:5′GATACCAGCACGACTGCTCA3'
MyD88	F:5′GTCTCCTCCACATCCTCCCT3'
	R:5′TAGACCAGACACAGGTGCCA3'
GAPDH	F:5′AGCAGTCCCGTACACTGGCAAAC-3′
	R:5′TCTGTGGTGATGTAAATGTCCTCT-3′

### Western blotting

The protein expression levels of molecules involved in TLR4-related signaling pathways in the study were determined by western blotting analysis, according to the standard manufacturer’s protocol. The antibodies of mouse monoclonal ApoA5, rabbit monoclonal toll-like receptor 4 (TLR4), mouse monoclonal myeloid differentiation factor 88 (MyD88), rabbit monoclonal nuclear factor kappa B (NF-κB) p65 were all purchased from Cell Signaling Technology, Inc. (Beverly, MA, USA), and the antibody of mouse polyclonal GAPDH was purchased from Jackson ImmunoResearch Laboratories (West Grove, PA, USA). The primary antibodies were all diluted at 1:1000, and the secondary antibody was diluted at 1: 3000. Immunodetection was performed with the ECL-Plus kit (Pierce Biotechnology, USA), and immunoblot signals were quantified using Quantity One Software (Bio-Rad).

## Results

### The expressions of ApoA5 gradually decreased with aggravated hepatic damage both in vitro and in vivo

**Fig. 1:**
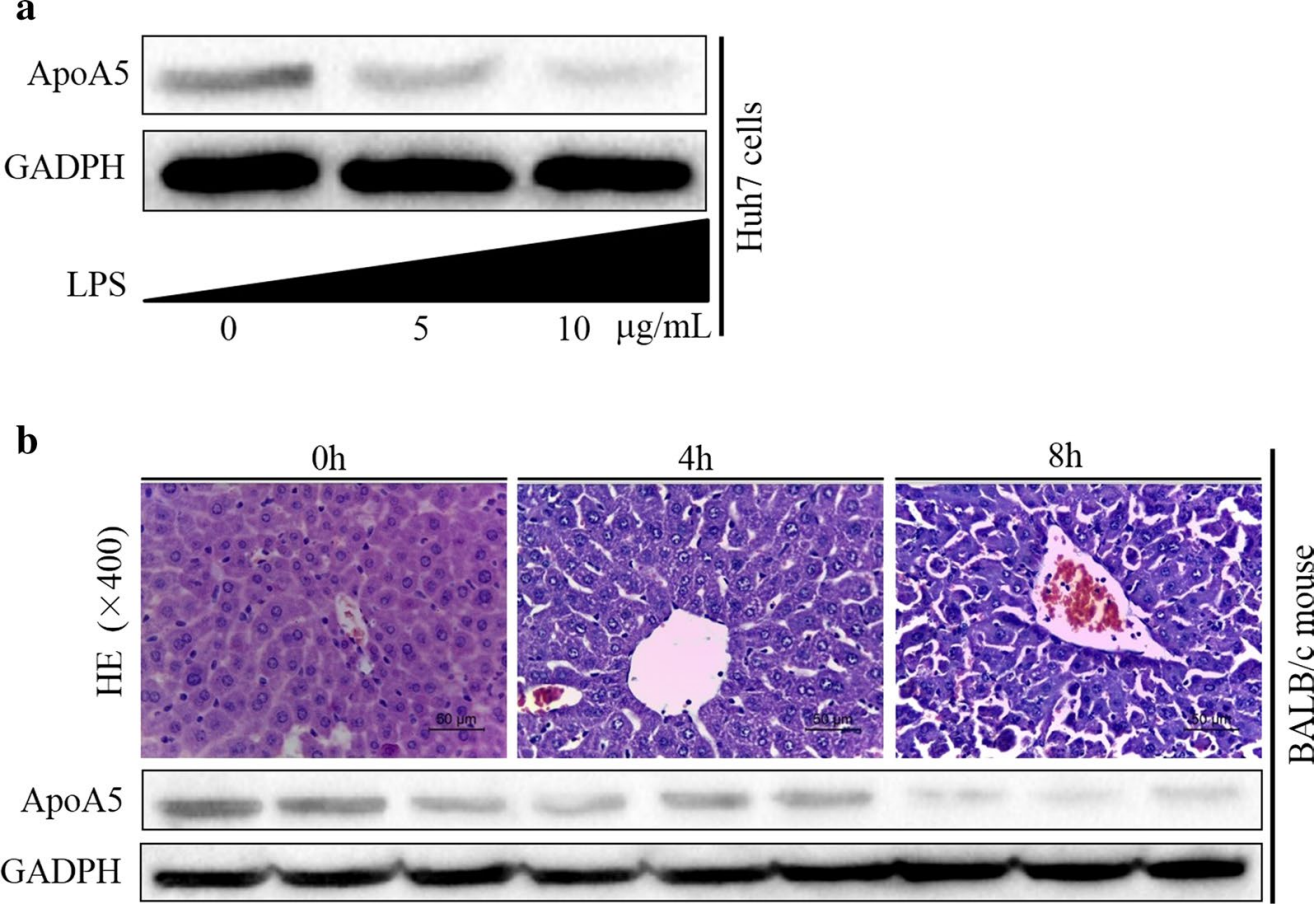
Dynamic change of ApoA5 protein levels in huh7 cells treated with different concentration of LPS (0 μg/mL, 5 μg/mL, 10 μg/mL) (**a**), and in the mice with LPS/d-GalN administration for different period of time (0 h, 4 h and 8 h) (**b**)

When huh7 cells were exposed to different concentrations of LPS, ApoA5 expressions gradually decreased (Fig. 1a). FHF mice were established with LPS/G-GalN for 8 h. The liver sections of different time points (0 h, 4 h and 8 h) showed increasing hepatocyte necrosis, destructive liver structure and inflammatory infiltration as treatment time went by, accompanied with downward trend of ApoA5 expressions (Fig. 1b). The results revealed that the expressions of ApoA5 gradually decreased both in vitro and in vivo as disease progressed.

### ApoA5 reduced cell apoptosis

**Fig. 2:**
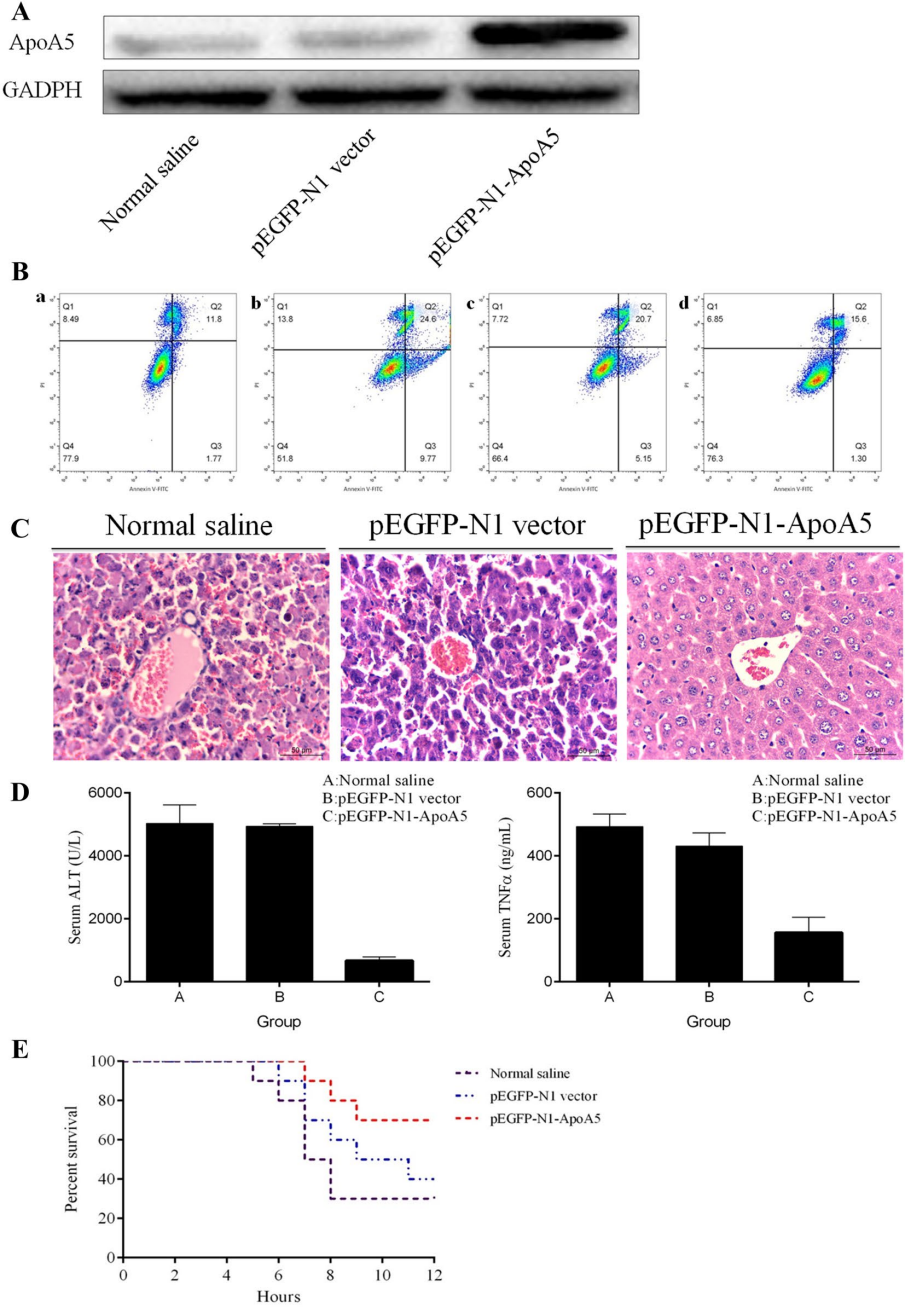
ApoA5 overexpression attenuated hepatic damage. **A** The protein expression level of ApoA5 in huh7 cells tranfected with pEGF-N1 ApoA5; **B** cell apoptosis analysis using Flow cytometry; **C** histology of liver sections stained with HE; **D** serum levels of ALT and TNF-α in the NS injected (group A), pEGF-N1 vector injected (group B) and pEGF-N1-ApoA injected (group C); **E** survival rate within 12 h after LPS/d-GalN administration in three groups

The levels of ApoA5 significantly increased in huh7 cells transfected with pEGF-N1-ApoA-5 (Fig. 2A). To evaluate the role of ApoA5 in hepatocyte damage, huh7 cells were stimulated with LPS (10 μg/mL) and cell apoptosis was analyzed within 12 h after LPS/d-GalN stimulation using annexin-V flow cytometry. As shown in Fig. 2B, cell death rate was 34.37% in cells only stimulated with LPS (Fig. 2B-b). And the cell death rate was 16.90% in cells transfected with pEGF-N1-ApoA5 before LPS addition (Fig. 2B-d), obviously lower than that in cells with LPS (16.90% vs. 34.37%) and in cells transfected with pEGF-N1-vector (16.90% vs. 25.85%), demonstrating that ApoA5 overexpression may reduce cell apoptosis.

### ApoA5 attenuated liver injury and improved the survival rate of FHF

The mice were randomly divided and were injected with normal saline (group A), pEGF-N1 vector (group B) and pEGF-N1-ApoA5 (group C), respectively. The HE- stained liver sections showed that LPS/d-GalN induced significant liver injury accompanied by hemorrhage, massive necrosis and inflammatory infiltration in the NS and empty control vector-injected mice (group A and C), but not in the mice pretreated with pEGF-N1-ApoA5 (Fig. 2C). The serum levels of ALT and TNFα at 8 h after LPS/d-GalN administration in group A and B were prominently higher than that in group C (Fig. 2D). We further analyzed the survival rates in three groups. As shown in Fig. 2E, the mice died at 5 h after LPS/d-GalN administration in group A and 7 h in group C. After LPS/d-GalN administration at 12 h, the survival rates were 30% for group A, 40% for group B, and 70% for group C, respectively. The finding further suggested that ApoA5 overexpression alleviated hepatic damage and improved the survival rate in FHF mice induced by LPS/d-GalN.

### ApoA5 inhibited TLR4-mediated NF-κB pathway

**Fig. 3:**
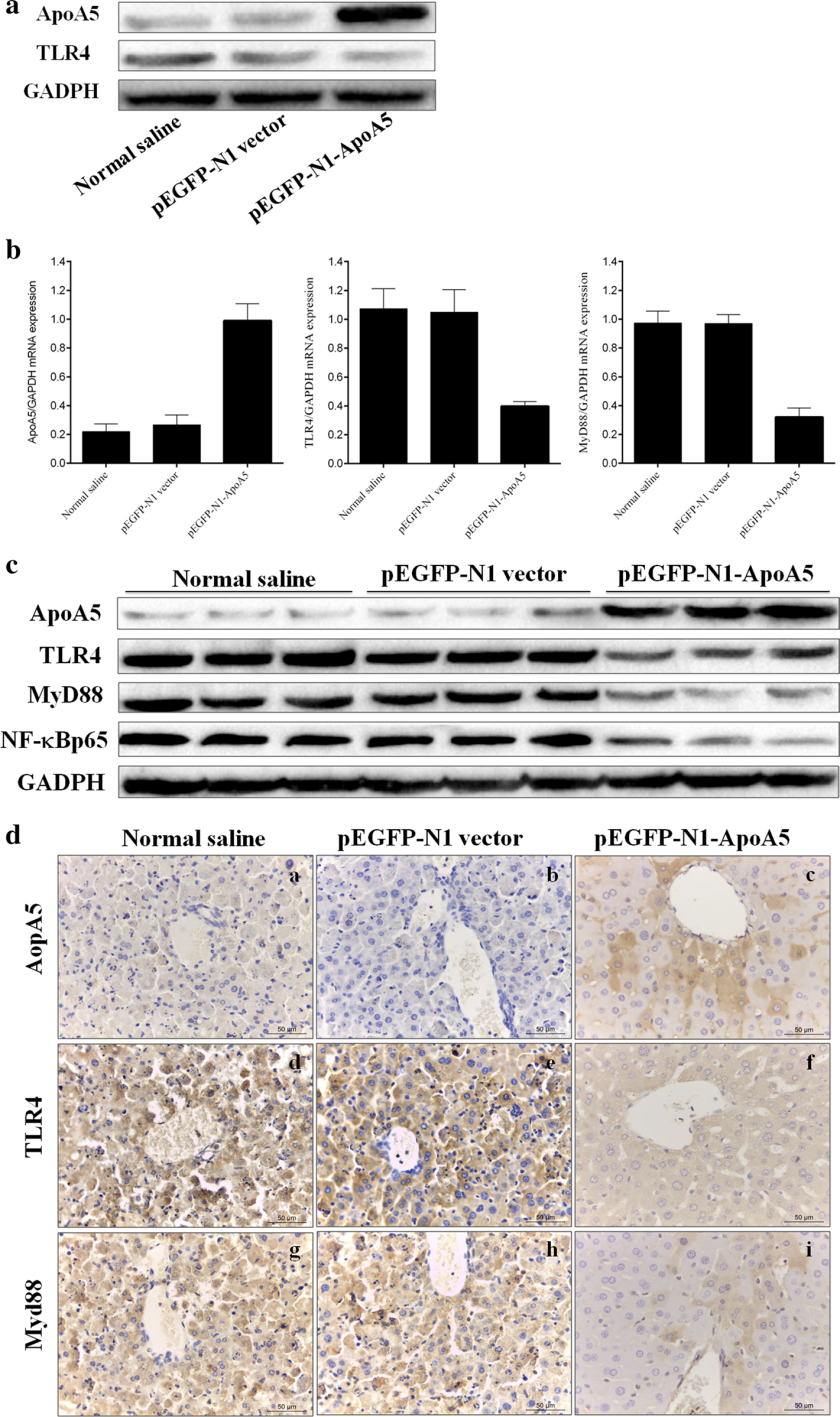
ApoA5 regulated key molecules expressions involved in TLR4 signaling pathway. **a** The protein expression levels of ApoA5 and TLR4 in huh7 cells transfected with pEGF-N1-ApoA5; **b** relative mRNA levels of molecules involved in TLR4 signaling pathway (ApoA5, TLR4 and MyD88); **c** effect of ApoA5 on the protein levels of ApoA5, TLR4 and MyD88 and NF-κBp65 after LPS/d-GalN administration by Western blot analysis; **d** assessment of immunohistochemistry for key molecules (ApoA5, TLR4 and MyD88) in liver tissue

It has been shown that TLR4 is identified as a receptor for LPS, and TLR4-related signaling pathway may mediate liver injury in FHF mice [18]. In present study, huh7 cells transfected with pEGF-N1-ApoA5 displayed elevated ApoA5 and decreased TLR4 expressions (Fig. 3a). In order to investigate the mechanism underlying the effect of ApoA5 in FHF mice, the hepatic expressions of TLR4 and MyD88 were assessed. The expressions of ApoA5, TLR4 and MyD88 were calculated relative to that of the endogenous control GAPDH. As shown in Fig. 3b, c, the mRNA and protein expressions of ApoA5 were distinctly higher, whereas the mRNA and protein expressions of TLR4 and MyD88 were lower in FHF mice pre-treated with pEGF-N1-ApoA5. Besides, pretreatment with ApoA5 was found to significantly decrease the protein expressions of downstream NF-κBp65. The protein expressions of ApoA5, TLR4 and MyD88 were in consistent with immunohistochemistry staining results (Fig. 3d).

### ApoA5 improved liver injury in a dose-dependent manner

**Fig. 4:**
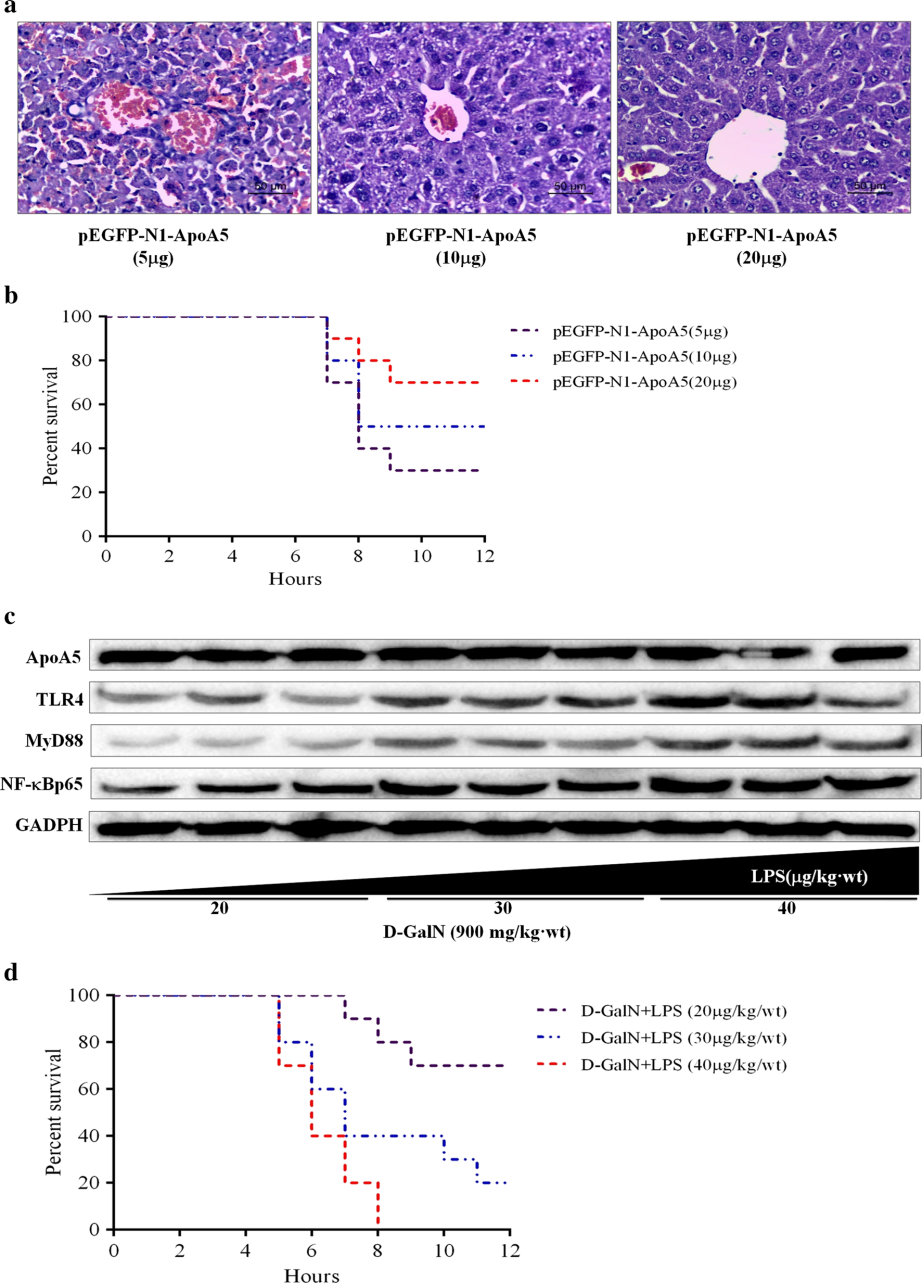
Both ApoA5 and LPS affect the severity of liver injury in a dose-dependent manner. **a** Liver histological damage was alleviated in a ApoA5-dose-dependent manner; **b** survival rate analysis in the mice pretreated with increasing concentrations of ApoA5; **c** the protein levels of ApoA5, TLR4 and MyD88 and NF-κB p65 in the mice confronted with d-GalN and increasing concentrations of LPS; **d** survival rate analysis in mice after injection of d-GalN and different concentrations of LPS

ApoA5 improved HE- stained liver section in a dose-dependent manner when increasing concentration of pEGF-N1-ApoA5 was injected to the mice by tail vein (Fig. 4a). Comparing the survival rates in the mice injected with different concentrations of pEGF-N1-ApoA5 (5 μg, 10 μg, 20 μg) before LPS/d-GalN administration, we found that the mice in three groups began to die at 7 h after LPS/d-GalN administration, but the survival rate in the mice with 20 μg pEGF-N1-ApoA5 was 70%, obviously higher than that in other two groups (50% for mice with 10 μg ApoA5 and 30% for mice with 5 μg ApoA5, Fig. 4b).

### ApoA5 attenuated liver injury within a certain range

The mice were pretreated with 20 μg pEGF-N1-ApoA5 prior to injection of d-GalN with different concentrations of LPS. As shown in Fig. 4c, ApoA5 expression remained unchanged, while protein levels of TLR4, MyD88 and NF-κBp65 elevated with the increasing concentrations of LPS (from 20 to 40 μg/kg). And the mice treated with the maximum LPS (40 μg/kg) died at about 5 h and reached 100% at 8 h after LPS/d-GalN administration, while the mice injected with the lowest concentration of LPS (20 μg/kg) showed the highest survival rate of 70% at the endpoint of our observation (Fig. 4d). The results revealed LPS induced liver injury in a dose-dependent manner. And the ApoA5 could attenuate liver injury within a certain range by inhibiting the TLR4-mediated NF-κB pathway. But the inhibitory effect of ApoA5 on TLR4-mediated NF-κB signaling pathway weakened with the increasing concentrations of LPS, and the protective role of ApoA5 against LPS/d-GalN-induced FHF was within a certain range.

## Discussion

Current interventions for FHF, including earlier recognition of the disease, emergency LT, critical care in the intensive care unit (ICU) and general support management out ICU, have contributed to the improved outcome of FHF in past decades [19]. However, there are still some patients dying from rapid progression and delayed LT owing to organ shortage. Hence, exploring other strategies to prevent waitlist mortality and increase the overall survival has been interesting. Targeting molecules that involve and determine the prognosis of the disease may be helpful for FHF therapy.

ApoA5 mediates lipid metabolism and energy generation, which is essential for liver generation and recovery in the process of FHF. The genetic sequence of ApoA5 was found in the liver, especially in regenerating liver [5]. Hendrik et al. [20] have reported that the newly formed membranes of regenerative cells is comprised of lipids, and ApoA5 might stimulate lipid uptake to promote the synthesis of membranes. But this speculation was a litter contradictory with the results they observed. The plasma concentration of apoA5 peaks at 6 h after partial hepatectomy, while mitosis initiates only 20 h later. Thus the function of ApoA5 in severe hepatic damage remained to be illustrated.

In present study, we observed the expression of ApoA5 gradually decreased as hepatic function got worse both in vitro (huh 7 cell) and in vivo (mouse model), demonstrating that ApoA5 is mainly synthesized in hepatocyte and its production may be influenced when the liver is under attack and the hepatic function is impaired. The ALT and TNF-α are considered as key indicators of liver injury. Our results showed that ApoA5 overexpression significantly decreased LPS/d-GalN-induced ALT and TNF-α productions in the serum. At the same time, mice histological analysis displayed ApoA5 alleviated LPS/d-GalN-induced liver injury. In addition, ApoA5 overexpression also reduced the apoptotic cells. These findings suggested a protective role of ApoA5 in LPS/d-GalN-induced liver injury.

Evidences have proved that TLR4, a member of TLR family, is the main receptor for the recognition of LPS and is quite vital in LPS-induced liver injury [21, 22]. It has reported that TLR4 upregulation functions in endotoxin-induced liver injury and decreases the mice survival rate after hepatectomy [21]. NF-κB is an important molecule located downstream of TLR4 signaling pathway and functions in inflammatory response [23]. TLR4 pathway in response to LPS can activate NF-κB and induce the production of pro-inflammatory cytokines, such as TNFα [24]. The mechanism of the combination of LPS and d-GalN induced liver failure partly relies on the activation of NF-κB signaling pathway. LPS triggers immune response via TLR4, following the activation of NF-κB pathway to release NF-κBp65 [25]. Then the released NF-κBp65 translocates to the nucleus and stimulates the transcription of inflammatory genes [26]. Thus, targeting the TLR4-mediated NF-κB pathway may be a way to alleviate LPS/d-GalN—induced liver failure. Previous study has proved that Baicalin may act as TLR4 antagonist and protect chicken from LPS-induced liver inflammation through down-regulation of TLR4 and NF-κB expressions [27]. Sea buckthorn polysaccharide extracts attenuated liver injury in LPS/d-GalN—challenged mice via inhibiting the TLR4-NF-κB signaling pathway [28]. Similarly, in this study we found ApoA5 pretreatment inhibited LPS/d-GalN- induced up-regulation of TLR4, MyD88 and NF-κBp65. These results indicated that ApoA5 exerted its hepatoprotective effect in mice by inhibiting TLR4- mediated NF-κB activation and it may be a potential therapeutic target for liver injury.

We further found that ApoA5 pretreatment could improve liver injury in dose-dependent manner. However, LPS induced liver injury in a dose-dependent pattern even if the mice were injected with 20 μg pEGF-N1-ApoA5, which demonstrated the fact that ApoA5 exhibited the protective effect against LPS/d-GalN-induced mice liver failure within a certain range. Although preclinical animal studies are essential for the development of diseases treatment, there may be some minor differences in the microenvironment between mice and humans, thus the exact role and mechanism of ApoA 5 in the process of human liver failure need further investigation and confirmation.

## Conclusion

In summary, this study demonstrated that ApoA5 had a protective effect against LPS/d-GalN-induced liver failure within a certain range by inhibiting TLR4-mediated NF-κB pathway, and ApoA5 might be a potential target for the prevention of FHF induced by LPS/d-GalN in mice.

## Data Availability

Not available.

## Abbreviations

FHF: fulminant liver failure
ALF: acute liver failure
ApoA5: apolipoprotein A-V
TLR4: toll-like receptor 4
MyD88: myeloid differentiation factor 88
NF-kBp65: nuclear factor-kBp65
ALT: aaminotransferase
TNF-α: tumor necrosis factor-α
LPS: lipopolysaccharides
d-GalN: d-galactosamine
